# Cervical cancer screening in Europe and Romania: a review of policies, progress, and persistent disparities

**DOI:** 10.25122/jml-2025-0099

**Published:** 2025-07

**Authors:** Gabriel Marian Saveliev, Valentin Nicolae Varlas, Madalina Piron-Dumitrascu, Nicolae Suciu

**Affiliations:** 1 Alessandrescu-Rusescu National Institute for Mother and Child Health, Bucharest, Romania; 2 Carol Davila University of Medicine and Pharmacy, Bucharest, Romania; 3 Filantropia Clinical Hospital of Obstetrics and Gynecology, Bucharest, Romania

**Keywords:** cervical cancer, HPV screening, Pap smear, HPV vaccination, Romania, Europe, cancer prevention, screening policy, public health disparities, cancer control strategy

## Abstract

Cervical cancer remains one of the leading causes of cancer-related death among women globally, despite being largely preventable through human papillomavirus (HPV) vaccination and regular screening. While many European countries have made significant progress in reducing incidence and mortality, Romania continues to report the highest rates within the European Union. This narrative review synthesized data from PubMed, Web of Science, Embase, and Google Scholar to evaluate cervical cancer screening policies across Europe, with a particular focus on Romania. The review included studies on HPV vaccination, cytology- and HPV-based screening, national program implementation, and public health strategies. Countries with organized, population-based screening programs and high HPV vaccine coverage, such as the Netherlands, Finland, and the UK, demonstrate lower incidence and mortality. In contrast, Romania faces persistent systemic barriers: limited public awareness, insufficient infrastructure, low screening participation (<20%), and suboptimal HPV vaccine uptake. Efforts to align national policies with WHO and EU cancer control strategies remain fragmented. Romania illustrates the deep disparities in cervical cancer prevention within Europe. Strategic reforms, including transitioning to HPV-based screening, expanding access to vaccination, enabling self-sampling, and enhancing public education, are critical. Integration into broader EU frameworks such as Europe’s Beating Cancer Plan may accelerate progress. While the tools for cervical cancer prevention are well established, Romania’s case underscores the need for systemic, context-specific interventions to reduce disease burden and promote equity across Europe.

## INTRODUCTION

Cervical cancer is a condition that can be prevented through vaccination and screening and can be treated effectively if it is detected in the early stages. However, it remains one of the most common forms of cancer and one of the leading causes of death among women worldwide [1]. In Europe, the incidence and mortality caused by cervical cancer have decreased significantly, but Romania continues to record the highest incidence and mortality rates in the entire region [2].

Cervical cancer is primarily caused by persistent infection with high-risk human papillomavirus (HPV) types, most notably HPV-16 and HPV-18 [3]. Globally, it is the fourth most common cancer among women, but incidence and mortality rates vary significantly within Europe due to differences in public health infrastructure, screening coverage, and vaccination uptake [4].

The World Health Organization (WHO) launched a global strategy in 2020 aiming for cervical cancer elimination by achieving three targets by 2030: 90% HPV vaccination coverage in girls, 70% screening coverage with a high-performance test, and 90% access to treatment for precancerous lesions and invasive cancer [3].

According to WHO, approximately 604,000 new cases of cervical cancer and more than 340,000 deaths from this disease are reported annually [3,5]. Most cases are diagnosed in women between the ages of 30 and 50, although the incidence can vary depending on geographical, socioeconomic factors, and access to screening and vaccination programs. Persistent infection with certain types of human papillomavirus, especially HPV-16 and HPV-18, is responsible for about 70% of all cervical cancer cases [4]. In the absence of preventive measures, such as HPV vaccination and regular screening (Pap smears and HPV testing), the disease can evolve rapidly, having a devastating impact on the health of women around the world [4,6].

In countries that have implemented effective HPV vaccination and screening programs, the incidence of cervical cancer has decreased significantly [7]. However, the lack of access to these essential resources in some regions continues to maintain high rates of the disease, underscoring the need for coordinated global interventions [8]. The link between HPV and cervical cancer emphasizes the importance of HPV vaccination, as well as regular screening by cervical Pap smear or HPV DNA tests, to be able to detect precancerous lesions early.

This review systematically examines existing literature and data regarding cervical cancer screening practices in Europe, with a particular emphasis on the Romanian context. A comprehensive search was conducted using major scientific databases including PubMed, Web of Science, Embase, and Google Scholar, employing keywords and combinations thereof: ‘cervical cancer screening’, ‘HPV screening’, ‘Pap test’, ‘HPV vaccination’, and ‘screening guidelines’.

Data extracted from the selected literature were synthesized narratively and structured thematically to facilitate a clear comparison of cervical cancer screening programs and their effectiveness, challenges, and recommendations for improvement in Romania.

## REVIEW

### Cervical cancer screening in Europe: current landscape

Screening for cervical cancer involves detecting precancerous changes in cervical cells, commonly using either cytology (Pap smear) or HPV DNA testing. European Guidelines for Quality Assurance recommend transitioning from cytology to HPV DNA testing for women aged 30 and above due to its higher sensitivity [9].

Countries like the Netherlands, Finland, and the United Kingdom operate organized, population-based programs with centralized registries and regular invitations. These systems demonstrate higher effectiveness and coverage (>70%). In contrast, countries with opportunistic screening, such as Germany and Austria, rely on individual initiative and healthcare provider recommendations, resulting in less consistent coverage [10].

Currently, the Gardasil vaccine, approved for both women and men, acts against HPV types 6, 11, 16, and 18, preventing precancerous lesions of the cervix and anus, as well as genital warts. The Gardasil 9 variant, authorized in December 2014, offers protection against nine types of HPV: 6, 11, 16, 18, 31, 33, 45, 52, and 58, as well as cross-immunity.

In countries that have implemented HPV vaccination programs on a large scale, a notable decrease in the incidence of cervical cancer and other HPV-related diseases such as genital warts has been observed [11]. In Australia, for example, which pioneered the introduction of HPV vaccination, the rate of cervical cancer has fallen by more than 50% among the vaccinated population, providing a strong example of the effectiveness of this program [12]. Also, data from other countries, such as the United Kingdom and Denmark, show a significant reduction in precancerous lesions and a lower incidence of cervical cancer in young women [13].

Despite EU efforts, there remains a wide gap in screening coverage and outcomes:

- Northern and Western European countries have well-established, high-coverage programs [14].
- Eastern and some Southern European countries, including Romania, Bulgaria, and Slovakia, struggle with implementation, outreach, and follow-up systems [4].

### Cervical cancer burden in Romania

According to GLOBOCAN 2020 data, Romania has an age-standardized incidence rate of approximately 22.6 per 100,000 women and a mortality rate of 10.8 per 100,000—among the highest in the EU [15]. Cervical cancer remains the second most frequent cancer in women aged 15-44 in the country.

**Figure 1:**
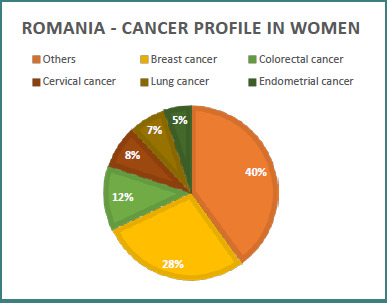
Relative distribution of cancer types in women (Romania)

**Figure 2:**
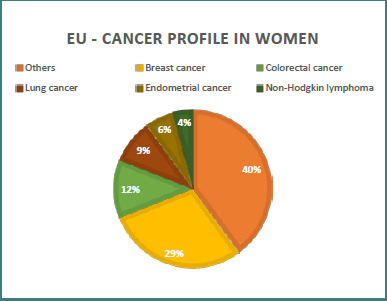
Relative distribution of cancer types in women (European Union)

Romania has the highest incidence and mortality rates for cervical cancer compared to other countries in the European Union (Figures 1 and 2). The incidence level of this type of cancer in Romania remains extremely high compared to the standardized average incidence rate in the European Union member states [3,5]. According to estimates by the Joint Research Center in 2020, 95,276 new cases of cancer were diagnosed in Romania. Among women, cervical cancer ranks third in frequency, after breast and colorectal cancer, a situation different from that at the European level, where breast, colorectal, lung, uterine body cancer, and lymphoma predominate [3,13].

In 2020, out of the total of 30,447 new cases and 13,437 deaths estimated in Europe due to cervical cancer, Romania registered 11.10% of new cases and 13.43% of deaths [16,17].

Romania initiated a national cervical cancer screening program in 2012, targeting women aged 25 to 64, offering free Pap testing every five years. However, participation remains critically low, estimated below 20% in many regions [18].

Factors contributing to poor uptake include insufficient public awareness, logistical challenges in rural regions, lack of a centralized registry and tracking system, and inadequate coordination between primary and secondary care levels [19].

Romania introduced HPV vaccination in 2008 but halted it due to low uptake driven by misinformation, limited public engagement, and cultural resistance. At the national level, there is currently a national vaccination campaign that ensures access to the vaccine free of charge for girls and boys between the ages of 11 and 18, and for women aged 19 to 45 years, it is partially reimbursed [20–22].

However, in many countries, including Romania, the HPV vaccination rate remains below the optimal level, for various reasons, such as lack of information, fear of adverse effects, vaccine hesitancy, or limited access to medical services. Improving these rates is essential to protect public health and reduce the number of preventable cancer cases.

### Systemic barriers in Romania and strategic interventions and policy alignment

Rural populations face significant challenges in accessing gynecological care. Many primary care units lack the infrastructure to perform Pap tests, and referral systems are weak. The absence of a unified health information system impedes follow-up care and data collection [23].

Also, poverty, low education levels, and cultural taboos around gynecological health reduce women's likelihood of participating in screening. Health literacy campaigns remain underfunded and inconsistently implemented [24].

The “Europe’s Beating Cancer Plan” launched in 2021 aims to address inequalities in cancer prevention and treatment by supporting member states in enhancing screening and vaccination programs [25]. Romania stands to benefit from these funds and technical guidance.

Currently, emerging evidence supports the feasibility of self-sampling for HPV testing, particularly for reaching underserved women. Pilot programs in Romania and other low-coverage EU countries show promising results in increasing participation rates [26].

NGOs and public agencies have begun implementing awareness campaigns through schools, churches, and local media. Integrating HPV vaccination into school health programs and improving provider training are considered high-impact strategies [27].

Romania represents a critical case study in the EU’s efforts to reduce cancer disparities. While the tools for cervical cancer prevention are well-established—HPV vaccination, HPV testing, and organized screening—effective implementation depends on systemic reforms.

Transitioning to HPV-based screening, establishing a national cancer registry, and integrating digital health solutions are essential steps forward. Public trust and engagement must also be rebuilt through transparent communication and culturally tailored education efforts.

Screening plays an essential role in the prevention and early detection of pre-invasive lesions, enabling the identification of cellular changes before they evolve into invasive forms of cancer. Early detection of these lesions allows for prompt intervention, significantly improving prognosis and reducing mortality associated with oncological diseases such as cervical cancer. Reforming the health system to facilitate access to screening, increasing education among the population, and improving prevention programs are essential to reduce the impact of cervical cancer in Romania. In order to improve cervical cancer screening in Romania, a systematic and comprehensive approach involving several areas is essential: health infrastructure, education, access to services, and logistical management.

## CONCLUSION

Europe is progressing toward cervical cancer elimination, but countries like Romania require intensified support and tailored strategies to overcome entrenched barriers. By aligning national efforts with EU initiatives and WHO goals, Romania has the potential to significantly reduce the burden of cervical cancer and contribute to the broader European public health agenda.
